# Appendicitis-related acute referrals to Children’s Health Ireland from Non-Specialist Paediatric Surgical Centres

**DOI:** 10.1007/s11845-023-03369-8

**Published:** 2023-04-21

**Authors:** Zakya Penny, Sami Abd Elwahab, Brendan O’Connor, Brian Sweeney

**Affiliations:** Department of General Paediatric Surgery, Children’s Health Ireland at Crumlin, Dublin, Ireland

**Keywords:** Appendicectomy, Appendicitis referrals, Paediatric appendicitis, Paediatric surgery

## Abstract

**Background:**

Acute appendicitis is the most common surgical emergency in children. Eighty percent of paediatric appendicectomies are performed by adult general surgeons on an annual basis. The remaining 20% are performed at Children’s Health Ireland (CHI) centres. Occasionally patients are transferred from Non-Specialist Paediatric Surgical Centres (NSPSC) for specialised pre-operative or post-operative care.

**Aim:**

To assess the rates of and characterise appendicitis-related referrals to CHI at Crumlin from NSPSC.

**Methods:**

A retrospective review of all appendicitis-related transfers to CHI at Crumlin between January 2020 and December 2021 was performed. Data relating to indications for transfer, referring hospital level, patient demographics, management, type of surgery, length of stay (LOS), and radiological studies were collected and analysed.

**Results:**

Seventy-two patients were transferred to CHI at Crumlin over the 2-year period. A total of 60.9% were male, mean age 9 ± 4.3 years, mean LOS 6.0 ± 2.2 days (range 1–30 days). Nineteen percent were under 5 years of age. Seventy-three percent were transferred from level 4 centres. Ninety-seven percent were transferred pre-operatively, 25% of those transferred pre-operatively had imaging in CHI confirming appendicitis. Fifty-five percent (40/72) of patients had pre-operative imaging performed. A total of 37.5% (15/40) confirmed complicated appendicitis. Twenty percent (8/40) underwent both ultrasound and computerised tomography (CT) at the referring centre. A total of 2.7% (2/72) were transferred with known co-morbidities. Ninety-two percent (66/72) underwent appendicectomy. Eight percent (6/72) were managed non-operatively (NOM) — 2 failed NOM, 2 underwent interval appendicectomy. Of those managed operatively, 76% (50/66) underwent laparoscopic appendicectomy, and 24% (16/66) were performed open.

**Conclusion:**

The majority of paediatric appendicectomies are performed at Non-Specialist Paediatric Surgical Centres. It is vital to maintain this working relationship so that specialist paediatric centres are available to provide care to complex paediatric patients.

## Introduction

Acute appendicitis is the most common surgical emergency in children. The incidence of appendicitis is approximately 1 per 1000 in the USA. Eighty-six cases of appendicitis per 100,000 children are estimated to occur annually in the USA, and this number is increasing. The overall lifetime risk is estimated to be around 8%, with a peak age during the teen years. Appendicitis is increasing in Hispanics, Asians, and Native Americans, whereas the rates in whites and African Americans have declined. There is a slight male preponderance (55–60%) [1]. It is estimated that more than 3000 paediatric appendicectomies are performed annually in Ireland. Eighty percent of paediatric appendicectomies are performed by adult general surgeons. Complicated appendicitis is more frequent in children under 5 years of age. The remaining 20% are performed at Children’s Health Ireland centres [2]. Occasionally patients are transferred from Non-Specialist Paediatric Surgical Centres (NSPSC) for specialised pre-operative or post-operative care. At present, there are no guidelines to regulate these transfers.

The management of appendicitis is becoming more standardised at many centres with the intention of limiting antibiotic duration, streamlining antibiotic therapies, limiting the number of hospital bed days and radiation exposure. However, across centres, there remains a wide variation in the management of appendicitis in the paediatric cohort.

The aim of this study was to identify indications for transfer to specialist paediatric centres, to determine the outcomes in this cohort, to determine whether diagnosis was confirmed in pre-operative referrals, the rate of radiological tests used after referral, the rate of operative and non-operative management, and overall length of stay.

## Methodology

A retrospective review of patients transferred to CHI at Crumlin from NSPSC was performed. Data was gathered via a three-pronged approach — the Hospital In-Patient Enquiry (HIPE) database, the hospital business manager database, and by audit of both the theatre log and of the surgical team handover records. All patients with appendicitis-related disease who were transferred from other centres were identified and examined. Data relating to patient demographics, length of stay (LOS), indications for transfer, referring hospital level, management (operative or non-operative), and radiological studies were collected for each patient. Data were anonymised so that the patients were not identifiable.

### Inclusion and exclusion criteria

All patients who were transferred from hospitals other than CHI at Crumlin with appendicitis-related disease between January 2020 and December 2021 were included. Patients who underwent appendicectomies as a secondary procedure whilst undergoing an alternative operation (such as in the case of a Ladd’s procedure) were excluded. Duplications were excluded.

### Outcomes measured

Outcomes measured included patient demographics, referring hospital levels, indications for transfer, overall LOS, operative and non-operative management, laparoscopic and open operations, post-operative complications, and radiological studies performed at the referring hospitals and at CHI Crumlin.

### Definition of level of hospital

Model 4 hospitals admit undifferentiated acute medical patients including tertiary referred patients. These level 4 hospitals have a category 3 or 3S intensive care unit (ICU) on site, an acute medical assessment unit (AMAU/AMU) which is open on a continuous basis (24 h, every day of the year) and an emergency department (ED) and includes a clinical decision unit (CDU) on site. A category 3 ICU is defined as patients requiring two or more organ support or needing mechanical ventilation alone. These ICUs provide one-to-one nursing care with a doctor present in the unit 24 h a day [3].

Model 3 hospitals admit undifferentiated acute medical patients. These level 3 hospitals have an AMAU and ED on site. This hospital has a category 1 or 2 ICU. Category 2 ICU is defined as patients requiring single organ support (excluding mechanical ventilation) such as renal hemofiltration or ionotropic support and invasive blood pressure monitoring. They are staffed with one nurse to two patients. Category 1 ICU is defined as ward-based care, where the patients does not require organ support.

Model 2 hospitals provide inpatients and outpatient care for differentiated, low-risk medical patients who are not likely to require full resuscitation.

Model 1 hospital include community or district hospitals [4].

### Statistical analysis

Descriptive statistics were used to analyse the results. Where appropriate, significance was measured using chi squared and Student *t*-tests. A *p* value of < 0.05 was considered significant. STATA V17 ™ was used.

## Results

Seventy-two patients were transferred to CHI at Crumlin over the 2-year period. A total of 60.9% were male, the mean age was 9 ± 4.3 years, mean LOS 6.0 ± 2.2 days (range 1–30 days). Nineteen percent were under 5 years of age. Seventy-three percent (50/72) were transferred from level 4 centres.

Ninety-seven percent were transferred pre-operatively, 25% of those transferred pre-operatively had imaging confirming appendicitis. In total, 40 patients had pre-operative imaging performed. A total of 37.5% (15/40) confirmed complicated appendicitis. Twenty percent (8/40) underwent both ultrasound and computerised tomography (CT) at the referring centre.

A total of 3/72 were transferred with known co-morbidities. One patient with a history of rhabdomyosarcoma cited as the indication for transfer was referred from a level 3 centre. Pre-operative imaging performed in two modalities at the referring centre confirmed acute uncomplicated appendicitis. An uncomplicated laparoscopic appendicectomy (AAST 2) was performed, the post-operative period was uncomplicated, the LOS was 4 days. One 16-year-old patient with a history of hypoplastic left heart syndrome was transferred from a level 3 centre. There was no cited indication for transfer. Pre-operative imaging at CHI Crumlin did not visualise the appendix. A laparoscopic appendicectomy was performed, the appendix appeared inflamed (AAST 1), histology confirmed acute appendicitis. The post-operative period was uncomplicated, the LOS was 3 days. One patient with a history of Von Willebrand Disease was transferred from a level 3 centre. There was no specific indication for transfer. No pre-operative imaging was performed. An open appendicectomy was performed for an acute uncomplicated appendicitis (AAST 2). The patient was commenced on a 5-day course of oral tranexamic acid on the advice of haematology, the overall LOS was 5 days.

Indications for transfer were documented in 14/72 (19.4%) patients; 6/14 (42.9%) were transferred with known complicated appendicitis on imaging. Two of the 14 patients (14.3%) were transferred post appendicectomy with bladder injuries following port insertion. Another two patients were post-operative — one with a small bowel obstruction (SBO), the other post an attempted appendicectomy which was abandoned on finding an inflammatory mass on laparoscopy. Two patients had failed conservative management. One patient was transferred as there were no beds available in the level 4 referring centre, and another one was considered too young at the age of two for management of simple appendicitis at a level 3 centre. There was no clearly documented indication for transfer for the remaining 58 patients.

Ninety-two percent (66/72) underwent appendicectomy — 76% underwent laparoscopic appendicectomy, 24% performed open. Eight percent (6/72) were managed non-operatively (NOM) — 2 failed conservative management and were readmitted within 3 months one requiring a laparotomy and adhesiolysis, the other with extensive intra-abdominal collections requiring percutaneous drainage via interventional radiology (IR). Two underwent interval appendicectomy.

Intra-operative grading of appendicitis using the American Association of Surgical Trauma (AAST) classified 40% (29/72) as grade 3 or higher [5].

Two patients were seriously ill requiring paediatric ICU (PICU) admission. One patient with AAST 4b appendicitis required a one-night stay in PICU post-operatively, with an overall LOS of 6 days. Post-operative targeted antibiotics resulted in a Clavien-Dindo score of 2, their post-operative course was otherwise uncomplicated. One 15-year-old patient required a peri-operative admission to PICU with a lactate of 5. The patient was transferred from a level 4 centre, pre-operative imaging was performed at CHI and confirmed four quadrant collections. A laparotomy and washout for complicated appendicitis (AAST 5) was performed, more than 1 l of pus was washed out. The patient remained in PICU for 7 days, not requiring inotropic support or invasive ventilation. An NG tube was placed for post-operative ileus, and TPN was provided for 3 days. Clavien-Dindo complication grade 4b.

Fifteen percent (10/66) suffered post-operative complications, 60% (6/10) collections, 10% (1/10) suprapubic port site infection, 10% (1/10) ileus, 10% (1/10) port site wound dehiscence, and 10% (1/10) SBO (Table 1). Nine percent (6/66) suffered grade 3–4b Clavien Dindo complications [6]. A total of 1/6 had omentum protruding from the port site, 1/6 SBO requiring laparotomy and adhesiolysis, 3/6 laparotomies for collections not amenable to drainage, and 1/6 collections — drained under IR guidance.

**Table 1:** Post-operative complications (10 patients)

Collections	60%
Suprapubic port site infection	10%
Ileus	10%
Port site wound dehiscence	10%
SBO	10%

## Discussion

The majority of cases were transferred pre-operatively with simple appendicitis, and only 6 cases were transferred with known complicated appendicitis on imaging. Eighty-one percent of patients were over the age of 5. Whilst no clear indication for transfer was cited, it is possible that in these cases, patients were transferred as the referring surgical team and/or anaesthetic team were not comfortable managing the paediatric patient [7].

More than half of all patients underwent pre-operative imaging, the majority of which was performed at CHI. On this basis, we would recommend pre-operative imaging for all patients in whom a diagnosis of appendicitis is suspected, prior to consideration for transfer. At present, there are only 13 paediatric radiologists nationally, the majority of whom (11.8) are designated to CHI centres. Whilst this would suggest the majority are pooled at designated centres and makes an argument for pre-operative imaging to be performed at the tertiary centre, consideration must be made for allocation of resources and time, given that CHI Crumlin now represents the only acute paediatric surgical centre in the country. The proposed model of care outlined by the Health Service Executive aims to treble the number of paediatric radiologists nationally in the coming years, with a view to maintaining specialist paediatric radiologists at centres outside Dublin [8]. Referral to a specialist centre should not be delayed by pre-operative imaging; however, if the diagnosis is uncertain, it is reasonable that an ultrasound is done prior to transfer when the patient is systemically well and is able to tolerate the test. The gold standard of investigation is ultrasound, with a recognised international overall rate of sensitivity of 97.9% and specificity of 91.7% in diagnosing paediatric appendicitis [9]. Of the patients in our cohort with pre-operative imaging (8/40) underwent both ultrasound and computerised tomography. In the absence of a paediatric radiologist at the referring centre or an uncertain first-line diagnostic test our recommendation for second-line imaging would be CT, this carries with it a 96.3% sensitivity and 98.8% specificity. MRI may be utilised if available, internationally this is noted at a specificity of 100% [10]. The overall risk of radiation exposure is estimated at 1 case of cancer over a lifetime for every 1000 people scanned. This incidence is estimated at 24% greater in those who underwent CT than those that did not; this incidence was higher in those who were exposed at a younger age and was greater for certain types of solid cancer. In all instances, the benefit must outweigh the risk and the decision to scan must be made on a case-by-case basis [11, 12].

The complexity of cases in our cohort and lengths of stay correlate with the severity of appendicitis, the majority of prolonged admissions related to patients with perforated appendicitis. Our data shows a trend towards extended lengths of stay in patients with perforated appendicitis, open procedures, and conservatively managed appendicitis. Thirty-one patients had a perforated appendicitis with a cumulative 278 bed days (range 4–29 days). LOS in those who underwent laparoscopic appendicectomy for an acute uncomplicated appendicectomy was up to 3 days compared to < 48 h internationally [13].

About 24% underwent an open appendicectomy; in most of these cases, this related to a large, fixed mass palpated at the time of examination under anaesthetic. Those who underwent an open appendicectomy were between the ages of 1 and 14 years old. International studies with a similar case mix quote a rate of open appendicectomy of up to 40% [14].

Our study identified that in over 80% of cases, the indication of transfer was not documented, neither in CHI noted nor the transfer letters. This poses a significant gap in the medical records for these patients. On talking to CHI staff, the reasons quoted for these gaps is that the indication relates to the patient’s age, and this varies greatly from one centre to another.

The study highlights the urgent need for ongoing engagement with anaesthesiology, adult surgery, and paediatric radiology in order to formulate a pathway for management of these cases. An exact cut-off point regarding age must be decided by consensus across these three governing bodies at the NSPSC nationally in order to establish a clear management protocol and referral pathway. In all cases of transfer to a specialist centre, a clear reason must be documented so that the deficiencies and needs of the referring centre can be addressed and improved upon, to reflect a change in policy and utilisation of resources. This will enable the specialist surgical centres to implement a safe standardised protocol for management, allowing for ongoing collaboration nationally, whilst preserving capacity at CHI.

## Limitations

This was a single-centre retrospective study. The database was incomplete with respect to the indication for transfer; the exact indication was cited for only 16 patients. Additionally, the number of cases included likely under-represents the total number that were transferred [2], highlighting the need for more thorough and efficient data management within the department/hospital.

## Conclusion

Ongoing collaboration between CHI and adult emergency general services will enhance the care of these children, reduce the travel burden on patients, protect CHI capacity, and allow for management of more complex subspecialised patients. It is imperative that patients be transferred as promptly as possible to paediatric specialist centres if the diagnosis is uncertain, if the patient is too young, too sick, or too complex. We aim to conduct a prospective study recording all transfers and their indications in order to formulate of a management pathway and improve patient care.

## Data Availability

Raw data is available on request.
